# Occurrence, distribution and risk assessment of phthalate esters in dust deposited in the outdoor environment of Yazd industrial park using Monte Carlo simulation

**DOI:** 10.1016/j.heliyon.2024.e37500

**Published:** 2024-09-08

**Authors:** Mohammad Hasan Ehrampush, Ehsan Abouee, Hossein Arfaeinia, Zahra soltanian, Mahdi Ghorbanian, Sahar Ghalehaskari

**Affiliations:** a Environmental Science and Technology Research Center, Department of Environmental Health Engineering, School of Public Health, Shahid Sadoughi University of Medical Sciences, Yazd, Iran; b Department of Environmental Health Engineering, School of Public Health, Bushehr University of Medical Sciences, Bushehr, Iran; c Department of Environmental Health Engineering, North Khorasan University of Medical Sciences, Iran; d Vector-borne diseases research center, North Khorasan University of Medical Sciences, Bojnoord, Iran

**Keywords:** Health risk assessment, Industrial park, Monte Carlo simulation, Phthalate esters, Outdoor dust, Yazd

## Abstract

In this study, the distribution of eight phthalate esters (PAEs), namely (dimethyl phthalate (DMP), diethyl phthalate (DEP), di-n-butyl phthalate (DBP), butyl benzyl phthalate (BBP), bis (2-ethylhexyl) phthalate (DEHP), and di-n-octyl phthalate (DnOP)) were examined across fifteen sampling stations in Yazd industrial Park. All the PAEs in dust deposited in the outdoor environment were analyzed using a Gas-mass chromatography (GC-MS/MS) device. Both probabilistic and deterministic approaches were utilized to assess the non-carcinogenic and carcinogenic health risks for adult occupational population groups. These risks were associated with three exposure pathways: inhalation, ingestion, and dermal exposure to six phthalates in the dust samples. The findings revealed, among the fifteen sampling stations, highest and lowest concentrations of the PAEs in dust deposited in the outdoor environment were observed in S8 and S6, with BEHP (326.21 ± 4.35) μg/g and DMP (0.00 ± 0.02) μg/g, respectively. The total hazard index (HI) values were below one in all samples, indicating that the combined non-carcinogenic health risk from exposure to phthalates via inhalation, ingestion, and dermal pathways is within acceptable levels in each studied area. The total cancer risk (CR) values for BBP across all exposure routes were consistently low, with magnitudes ranging from 10- x 10^−15^to 10 x 10^−11^. The order of cancer risk from phthalate exposure in outdoor environments was ingestion > dermal > inhalation. The sensitivity analysis (SA) results indicated that the influential parameters in the carcinogenic risk in adult occupational population groups were concentration for inhalation and dermal pathways, as well as ingestion rate for the ingestion pathway. The result of this study provides new insight in to PAEs pollution and risk assessments related to the dust deposited in the outdoor environment of industrial Park. Furthermore, this finding is beneficial to the controlling the exposure and promoting steps to reduce PAEs contamination and manage health in the industrial area.

## 1 Introduction

Human activities have played a significant role in elevating levels of heavy metals, plastics, and phthalate esters (PAEs). Global plastic production currently amounts to 368 million tons annually, with phthalate ester consumption reaching 6–8 million tons in 2017 [1]. The unsustainable practices in producing, using, and disposing plastics have exacerbated global plastic pollution. This pollution accelerates the breakdown of plastics into PEs, which poses significant risks to both environmental and human health [2]. In recent decades, PAEs have recognized as a group of emerging air pollutants. because they are emitted to the atmosphere as particulates and gases and difficult to degrade and extensively persist in air [3]. These compounds are extensively used in producing various commercial materials, such as rubber, cellulose, styrene, and other products. PAEs can leach from polymer matrices under physical stress conditions, releasing them into the packaging contents or the environment, posing significant risks to human health and the environment [[4], [5], [6], [7]]. [8],. Recently, there has been growing concern regarding the potential for exposure to this pollution to cause endocrine disruption and carcinogenic effects in both humans and animals [3]. Six groups of phthalate esters including dimethyl phthalate (DMP), diethyl phthalate (DEP), di-n-butyl phthalate (DnBP), di (2- di(2-ethylhexyl) phthalate (DEHP), butyl benzyl phthalate (BBP) and di-n-octyl phthalate (DnOP) are classified as a pollutants Priority and dangerous according Environmental Protection Agency (EPA) category [9].Chronic exposure to PAEs has been linked to various types of cancer, including breast cancer in women and prostate cancer in men, disorder in the synthesis and activity of male hormones (anti-androgenic), thyroid gland dysfunction, sterility, teratogenic and mutagenic activity. PAEs have also been implicated in children's hyperactivity, obesity, liver damage, increased oxidative stress, allergies, and asthma [10,11] [12],.

As a developing country, Iran's industrial parks have become significant sources of environmental pollutants. It has been more than two decades since these industrial parks were established to foster industrial growth and implement policies for population redistribution. The primary objective is to optimize natural resource utilization, prevent the uneven concentration of industries and industrial density in cities, and align industrial development with environmental protection, which has been a priority for authorities. Industrial park was an important carrier of trace hazardous substances such as micro plastic, poly aromatic hydrocarbons and PAEs from various sources [13].

Yazd province, particularly Yazd city, possesses considerable industrial capabilities and potential, with industry now recognized as a key driver of growth and development. The province hosts approximately 2172 licensed production and industrial units, manufacturing over 730 products and industrial goods. Textiles remain the dominant and longstanding industry in the province, supported by more than 483 production units. Furthermore, in 2016, global production exceeded 5.4 million tons of synthetic fibers, highlighting concerns over their environmental impact as they enter the aquatic ecosystems through fabric washing processes. Settled dust is one of the most important indicators reflecting the state of air pollution in the urban environment and one of the important sources of pollution that have a significant impact on ecological quality, which is the reason for having a capacity in maintenance the microplastics and phthalate, also the settled dust is one of the important sources of entering the particulate matter to the atmosphere [14,15], [16].

Several studies on PAEs have been conducted globally, examining their presence in various environmental matrices, including water, sediment, dust, soil, and biota, including aquatic and terrestrial wildlife, as well as in industrial park dust, which can serve as environmental monitoring tools [16] [17], [18,19], [[20], [21], [22], [23]], [[24], [25], [26]],.Settled dust is one of the most important indicators reflecting the state of air pollution in the urban environment and one of the important sources of pollution that have a significant impact on ecological quality, which is the reason for having a capacity in maintenance the microplastics and phthalate, also the settled dust is one of the important sources of entering the particulate matter to the atmosphere [27]. Contaminated dusts from industrial park area can be threaten human health via three exposure pathways: ingestion of dust, inhalation of dust and dermal adsorption of dust particles [28]. In particular, PEs in industrial park dust can serve as valuable environmental monitoring tools. Therefore, it is crucial to investigate the concentrations of PEs, and the degree of exposure through the three predominant routes (outdoor dust) in different environments is crucial. Hence, studying PAEs concentration and the degree of exposure via the three predominant routes of outdoor dust in different environments is important Therefore, the present research focus to (1) determine the concentration of PAEs in outdoor dust from 15 stations industrial park (2) spatial distribution of PAEs in study area and (3) evaluate the human health risk assessment using Monte Carlo simulation for adult occupational population. To the best of our knowledge, the hazard index (HI) and carcinogenic risk (CR) methods were employed to evaluate the non-carcinogenic and carcinogenic health risks for adults.

## 2 Materials and method

### 2.1 Study area

**Fig. 1:**
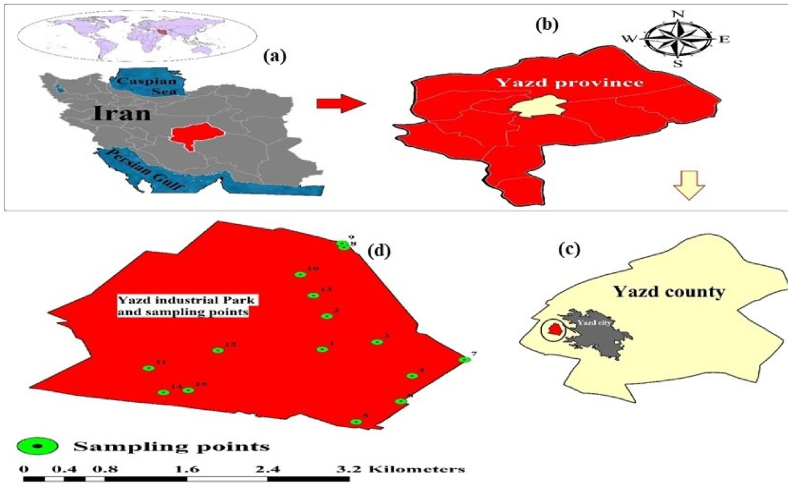
Location map and sampling points of the study area. a) Iran, b) Yazd Province, c) Yazd county and d), Industrial park.

Yazd province and especially Yazd city has many abilities and potentials in the industry sector and today industry is considered as one of the most important factors of growth and development of this city. The province has approximately 2172 production and industrial units with operating licenses, which produce more than 730 types of products and industrial goods (Fig. 1(a–c)).

### 2.2 Sample collection

The focus of this study was on settled dust in the external environment of the industrial city of Yazd. The sampling regularly and systematically was done.15 sampling stations from the outer environment of the industrial city were selected to measure phthalate esters. Of these 15 stations, three stations, one in the northern area and two stations in the southern area of the industrial town, the closest residential area to the industrial town, were selected as control stations (Fig. 1)d)). In total, 45 samples were collected, each with two repetitions. The number of sampling stations is chosen based on numerous studies conducted on the assessment of phthalate ester concentrations in various areas, as seen in recent studies worldwide. Additionally, the minimum number of samples required for statistical modeling has been selected. The number of sampling stations was also chosen in a method that covers the entire desired surface based on the study area's size. This study used 60 mL colorless glass containers for sample collection. Prior to sampling, each container was thoroughly washed with soap and water, followed by two rinses with distilled water. The cleaned containers were then placed in the oven at 180 °C for 1 h. Immediately after, the containers were removed from the oven, and their lids were secured to prevent contamination from dust. The lids remained closed until the time of sampling. Simultaneously, internal dust sampling was conducted to assess microplastics. Dust samples were collected from surfaces using a brush and a glass plate and transferred to glass bottles. Each station varied from 2 to 5 g according to its specifications. To prevent the transfer of contamination, after each sampling, the brush and the glass plate were washed first with normal water and then with distilled water [29]. After coding, the samples were kept in a dry environment until transfer to the laboratory.

### 2.3 Reagents and materials

Individual neat crystal phthalates standards, including Dimethyl phthalate (DMP), diethyl phthalate (DEP), di-n-butyl phthalate (DBP), bis(2-ethylhexyl) phthalate (BEHP), butyl benzyl phthalate (BBP) and di n-octyl-phthalate (DNOP) were obtained from Sigma. the purities of phthalate standards and isotope internal standards were above 98 %. Methanol and acetonitrile were purchased from Thermo Fisher (Waltham, MA, USA). Dichloromethane, n-hexane, sodium sulfate, sodium chloride and sodium hydroxide were obtained from Merck (Waltham, MA, USA) with purity of above 95 %. Ultrapure water was prepared by Milli-Q® Gradient A10 (Merck Millipore, Burlington, MA, USA).

### 2.4 Sample preparation procedure

The samples are freeze-dried for 72 h in the laboratory. Then samples passed through a 0.5 mm sieve until they are completely homogenized. Then we picked up 10 g of the homogenized dry sediments and the extraction process out using the solvent extraction method was carried. The extracted samples were placed in glass vials washed by acetonitrile and covered with suitable aluminum foils. Then it was stored at −20° until further processing analysis. Finally, 4 μL of this extracted solution were injected into the GC device and the concentration of these compounds was determined [28].

### 2.5 Instrumentation and chromatographic conditions

In this study, 5 g of the sample was tacked and extracted using hexane dichloromethane solvent then cleaned and analyzed in the following way. All the PEs investigation was performed using a Gas-mass chromatography (GC-MS/MS) device manufactured by Agilent (USA model 7890 equipped with MS model 5975 Split/Spitless) input which has a quadrupole type mass spectrometer. A polydimethylsiloxane capillary column (HP-5 MS (5%phenl)-95 %) (30 m × 0.25 mm × 0.25 μm) gas chromatography from Agilent made of silica was applied to separate PEs. Perfluorotributylamine (PFTBA) was used for mass spectrometer calibration. Single ion monitoring (SIM) analysis was used for each of the target species. Oven temperature was set initially at 70 °C (hold for 1 min), then increased to 300 °C at 10 °C/min (hold for 10 min).

At 310 °C, temperature was maintained for 5 min. Helium with a purity of 99.99 % used as a carrier gas in a constant flow of 1 mL/min and the injection volume 1 μL with an autosampler in spitless mode to optimize the separation and resolution between the chromatogram peaks obtained from different temperature programs for the column and different carrier gas inlet flow rates. The GC was interfaced by a heated transfer liner (290 °C) to the mass spectrometer in electron ionization mode with an electron energy of 70 eV. Inlet temperature was 290 °C and inject volume was 1 μL. The source and quadruple temperatures were kept at 230 and 150 °C, respectively [30]. Data processing was done by MSD Chem Station software from version E.January 02, 1177. The parent ion with the highest frequency, as listed in Table S1, was selected to increase the device's sensitivity and accurately analyze the composition of phthalates using the SIM program.

### 2.6 Quality assurance and quality control

Three standard solutions, including 500, 1000, and 1500 ppb were prepared by diluting the main stock solution using PAEs and injected into the device at the same volume of the sample size [31]. The calibration curve of the 3 PAEs was plotted. The correlation coefficients (R^2^) of the calibration curve for the dust samples at three concentrations ranged from 0.990 to 0.996. The limit of detection (LOD) for all the PEs was from 0. 1–2.11 ng mL−1 (both hands) and the limit of quantitation (LOQ) in S/N from 10 is in the range of 0.34–6.97 ng mL−1(table S-2). The recovery rate in the present study varied from 89.7 % to 107.3 % for these PEs according to the proposed range of the SANTE guideline (from 0.1 % to 19.6 %). The repeatability (RSDr) is calculated from the results of four replicate experiments in a single day of standard 500, 1000, and 1500 ppb μg/L. Relative standard deviation (RSD) values for DMP, DEP, IBP, DBP, BBP, BEHP and DOP were 9.4 %, 6.5 %, 3.4, 2.2,5.2, 1.9 and 2.7 %, respectively (table S-3). For improving the device sensitivity and examining the PAEs compounds more accurately, according to the Single ion monitoring program, the selected ion with the maximum frequency was selected as presented in (table S-3).

### 2.7 Standard chromatogram

To affirm the standard chromatogram for DMP, DEP, IBP, DBP, BBP, BEHP and DOP, we injected standard solutions into the GC-MS/MS device. because these standard chromatograms were necessary to identifying and confirming the presence of specific PAEs in the samples. Subsequently, we injected the dust samples into the GC-MS/MS device, allowing us to generate chromatograms for each sample, as shown in Fig. 1S. This stage allowed us to analyze the PAEs present in the dust samples.

### 2.8 Health risk assessment _ deterministic

In this study's, the Health Risk Assessment (HRA) constitutes a crucial aspect, focusing on the evaluating both non-carcinogenic and carcinogenic risks associated with exposure to phthalates present in dust samples collected from the industrial city of Yazd. The HRA utilizes a comprehensive set of exposure assessment equations to estimate the potential health risks arising from dermal, ingestion, and inhalation pathways [32]. The concentration of phthalates in the dust samples, along with factors such as skin adherence, exposure frequency, and duration, forms the basis for calculating the Average Daily Dose (ADD) through each exposure route. The Hazard Quotient (HQ) is then determined as the sum of the ADDs normalized to the Reference Dose (RfD) for each phthalate. The HI consolidates the individual HQs, offering a holistic perspective on the cumulative non-carcinogenic health risks. The study's innovative approach integrates environmental and exposure data to, providing a comprehensive evaluate of the potential health impacts associated with phthalate exposure in the industrial city's ambient environment [33].

#### 2.8.1 Non-carcinogenic risk assessment

In the context of the study on dust samples collected in the Industrial Park of Yazd City, the non-carcinogenic risk assessment is conducted using specific exposure equations. The focus is on phthalates, and the health risk is assessed through the following equations [34] [35], [36],:

Eq (1) $${\text{ADD}}_{\text{inh}}=\frac{\;C_{s\;}{\times \text{IR}}_{\text{inh}}\times \text{EF}\times \text{ED}}{\;\text{BW}\times \text{AT}\times \text{PEF}}$$

Eq (2) $${\text{ADD}}_{\text{der}}=\frac{\text{Cs}\times \text{SA}\times \text{AF}\times \text{ABS}\times \text{EF}\times \text{ED}\times 10^{-6}}{\text{BW}\times \text{AT}}$$

Eq (3) $${\text{ADD}}_{\text{ing}}=\frac{\text{Cs}\times \text{IRing}\times \text{EF}\times \text{ED}\times 10^{-6}}{\text{BW}\times \text{AT}}$$

Eq (4) $$\text{HQ}=\sum \frac{{\text{ADD}}_{j}}{\text{RfD}}$$

Where;

Cs: Concentration of phthalates in dust samples (μg/g)

IR_ing_: Ingestion rate for dust (mg/day)

EF: Exposure frequency (days/year)

ED: Exposure duration (years)

PEF: Particulate emission factor (m³/day)

AT: Averaging time (days)

BW: Body weight (kg)

SA: The dermal exposure area (cm^2^)

AF: Dermal adherence factor (unitless)

ABS: Fraction of material absorbed through the skin (unitless)

IR_inh_: Inhalation rate for dust (m³/day)

RfD: Reference Dose for phthalates (mg/kg/day)

The Hazard Index (HI) was utilized to assess non-cancer risks, as expressed by Eq (5):

Eq (5) $$HI=\sum {\text{HQ}}_{j}$$

In this equation, the Hazard Index is determined as the summing the individual Hazard Quotients (HQj), where each quotient represents the ratio of the Average Daily Dose (ADD) for a specific exposure pathway to its corresponding Reference Dose (RfD). In this research, aligning with the established reference [35]. it's noteworthy that the Reference Dose (RfD) applied in this study remains consistent across three distinct exposure pathways. This unified RfD encompasses the oral reference dose (Rf Doral), the inhalation reference dose (RfDinh), and the dermal adsorption reference dose (RfD dermal). Mathematically, the formulation for RfD dermal is expressed as RfD dermal = Rf Doral × ABSGI, where ABSGI represents the fraction of the pollutant absorbed in the gastrointestinal tract. Remarkably, ABSGI is considered to be equivalent to one [[35], [36], [37]]. In risk assessments, the term 10-6 is often employed as a conversion factor to express exposure levels and risks on a per-person, per-day basis over a lifetime. This factor is used to convert micrograms (μg) to milligrams (mg) and is associated with the concept of one-in-a-million risk. Specifically, in the exposure equations provided (e.g., Eqs Eq (2), Eq (3)), 10^−6^ is utilized to convert certain quantities to milligrams per kilogram per day (mg/kg/day), allowing for a consistent and comparable expression of risk across different exposure pathways [33]. Table 1 presents the key parameters utilized in the Health Risk Assessment for adults, with corresponding units and specific values.

**Table 1:** Parameters for deterministic health risk assessment in adults exposed to phthalate.

Parameter	Unit	Adult	Reference
IR_ing_	mg/day	50	36
IR_inh_	m³/day	15.7	37
EF	days/year	300	Present study
ED	Years	30	Present study
PEF	m³/Kg	1.36 × 10^9^	38
AT	Days	365	Present study
BW	Kg	70	Present study
SA	cm^2^	4000	39
AF	Unitless	0.07	38
ABS	Unitless	0.001	39
RfD	mg/kg/day	DMP:10	37
		DEP: 0.8	40
		DIBP: 0.512	41
		DnBP: 0.1	
		BBP: 0.2	
		DOP: 0.4	
CSF	mg/kg/day	BBP: 0.0019	42

#### 2.8.2 Carcinogenic risk assessment (deterministic approach)

In contrast to the comprehensive non-carcinogenic risk assessment, the carcinogenic risk assessment specifically targets the compound Benzyl Butyl Phthalate (BBP). The evaluation employs specific exposure equations developed to quantify potential carcinogenic risks associated with to BBP exposure via ingestion, inhalation, and dermal pathways. The exposure equations utilized in the carcinogenic risk assessment are as follows [36,37] [38], [39,40],**:**

Eq (6) $${\text{LADD}}_{\text{inh}=}\;\frac{\text{Cs}\;}{\text{AT}\times \text{PEF}}\;\left(\frac{\text{IRinh}\;\left(\text{child}\right)\times \text{EF}\;\text{child}\times \text{ED}\;\text{child}}{\text{BW}\;\text{child}}⁺\;\frac{\text{IRinh}\;\left(\text{adult}\right)\times \text{EF}\;\text{adult}\times \text{ED}\;\text{adult}}{\text{BW}\;\text{adult}}\right)$$

Eq (7) $$\text{LADD}\_\left(\text{der}\right)=\frac{\text{Cs}\times \text{ABS}\times \text{EF}\times 10^{-6}}{\text{AT}}\;\left(\frac{\text{SA}\;\text{child}\times \text{AF}\;\text{child}\times \text{ED}\;\text{child}}{\text{BW}\;\text{child}}⁺\frac{\text{SA}\;\text{adult}\times \text{AF}\;\text{adult}\times \text{ED}\;\text{adult}\;}{\text{BW}\;\text{adult}}\right)$$

Table 2 outlines crucial parameters for adults in the HRA, including units and values. Eq (9) represents the Cancer Risk (CR) calculation in the context of carcinogenic risk assessment.

Eq (9) CR = LADDj × CSF

Where;

**Table 2:** Model parameters and their distributions for risk simulation and probability distribution.

Parameter	Abbreviation	Unit	Distribution	Value	Reference
Exposure Frequency	EF	d/y	Point	300	[43]
Exposure Duration	ED	y	Point	30	[40]
Average Time	AT	d	Point	900	
Particulate emission factor	PEF	m³/Kg	Point	1.36 × 10^9^	[41]
Fraction of material absorbed through the skin	ABS	Unitless	Point	0.001	[34]
Adherence Factor	AF	Unitless	Point	0.07	
Body Weight^a^	BW	kg	Log-normal	LN (68.9,8.9)	IRIS EPA
Ingestion Rate^b^	IR	L/d	Log-normal	LN (1.4, 1.56)	[42]
Inhalation Rate^b^	IR	m^3^/day	Log-normal	LN (32.73, 1.14)	[43]
Surface Area^b^	SA	cm^2^	Normal	N (20700, 3440)	[44]
Reference dosage of oral ingestion^b^	RfD_oral_	μg/(kg-day)	Point	Table 1	IRIS EPA
Reference dosage of dermal permeation^b^	RfD_dermal_	μg/(kg-day)	Point		
Cancer slope factor	Csf	(kg-day)/μg	Point	BBP (0.0019)	[ 45]

a For log-normal and normal distributions, the mean and standard deviation are represented as LN (mean, std. dev.) and N(mean, std. dev.).

b Adapted from USEPA.

**CR (Cancer Risk):** It signifies the potential risk of cancer associated with exposure to a specific compound, in this case, Benzyl Butyl Phthalate (BBP).

**LADDj (Lifetime Average Daily Dose):** It is the cumulative exposure dose over a lifetime, considering various exposure pathways (dermal, inhalation, ingestion) for a specific individual.

**CSF (Cancer Slope Factor):** This factor represents the slope of the dose-response curve and is specific to the carcinogenicity of the compound. It quantifies the increase in cancer risk per unit increase in exposure.

It is important to emphasize that this study, conducted within an occupational environment, in the context of this study conducted in an occupational environment, the exposure assessment is specifically tailoring the exposure assessment to adult populations engaged in industrial activities. Given the nature of the setting, children are excluded from the exposure assessment, with their exposure assumed to be zero, reflecting the study's occupational focus. This targeted approach ensures a focused evaluation of cancer risks associated with BBP exposure among the adult occupational population in the Industrial Park of Yazd.

#### 2.8.3 Probabilistic HRA approach by monte-carlo simulation

In the context of public health risk assessment, conventional methods often rely on mean, conservative, or worst-case values to calculate point estimates of risk, presuming them to be protective of public health. However, this approach has notable limitations, including ambiguity regarding the degree of conservatism, the inability to quantify the uncertainty associated with the final risk estimate, and the use of unrealistic maximum values for certain input variables. To address these shortcomings, a Monte Carlo simulation technique is employed in this study, offering a more robust and comprehensive approach to risk assessment [41]. The Monte Carlo method selects parameter values from probability distributions fitted to input data, enabling the calculation of point estimates and the distribution of exposure and risk. Through repeated iterations of the calculation process, statistical indicators such as average, minimum, maximum, standard deviation, and percentiles are generated, providing a more nuanced understanding of the variability and uncertainty inherent in the risk assessment. This technique enhances the reliability and value of the results compared to conventional point estimate methods, as it considers a wide range of potential sources of uncertainty, including sampling methods, experimental procedures, laboratory equipment, model structure, and human factors. Furthermore, uncertainty analysis, employing methods such as Spearman rank-order correlation, is utilized to assess how variations in input parameters influence the uncertainty of risk estimates, specifically focusing on Lifetime Cancer Risk (LTCR) and Hazard Quotient (HQ) as response variables in the models. A Monte Carlo simulation with a large number of replicates (e.g., 10,000 replicates) is conducted using Oracle Crystal Ball® (version 11.1.34190) to characterize the uncertainty and variability of input parameters in LTCR and HQ calculations, thereby providing a comprehensive understanding of health risks associated with exposure to phthalates [42]. Table 2 outlines model parameters and their respective distributions for risk simulation and probability distribution.

### 2.9 Statistical analysis

The Statistical Package for Social Sciences (SPSS, Windows Version 18, and Chicago, IL) was used for statistical analysis with significance defined as p = 0. 05. The normality of data was investigated using the Shapiro-Wilk test. Also, the analysis of data was used by ANOVA and *t*-test. Statistical differences among dust-phase phthalate levels in different locations were evaluated using Wilcoxon tests for paired samples. The correlations between concentrations of different compounds were evaluated using Spearman's rank correlation analysis.

## 3 Results and discussion

### 3.1 PEs concentrations in dust deposited in the outdoor environment of Yazd industrial park

The PAEs concentration of dust deposited in the outdoor environment of Yazd industrial were investigated and analyzed across the fifteen-sampling station of the study area during 2023. The corresponding PAEs concentration levels in the road dust samples from the three areas are listed in Table 3. Based on the results, the maximum and the minimum concentration of the PAEs in the dust deposited in the outdoor environment across the fifteen sampling stations to S8 and S6 with 326.21 ± 4.35 μg/g and 0.00 ± 0.02 μg/g, respectively. Variations in the concentration of PEs were observed across the sampled locations in of Yazd Industrial Park across the sampled locations. The highest level of PAEs (BEHP) was found in outdoor environment from location S6, with a mean value of 24.24 ± 83.564 μg/g.

**Table 3:** Concentrations (μg/g) of PEs in in dust deposited in the outdoor environment of Yazd industrial Park.

name		concentration Compound (ppm)				
		min	max	mean	SD	Allowable concentration*
**DMP**	R1	0.000	2.387	0.305	0.631	0.02
	R2	0.000	3.403	0.325	0.868	
	R3	0.000	1.820	0.333	0.529	
**DEP**	R1	0.017	0.162	0.041	0.038	0.071
	R2	0.075	0.242	0.128	0.050	
	R3	0.050	1.430	0.178	0.349	
**DBP**	R1	0.021	0.766	0.146	0.220	0.081
	R2	0.054	0.276	0.118	0.065	
	R3	0.060	0.450	0.116	0.098	
**IBP**	R1	0.088	13.151	1.525	3.385	
	R2	0.234	1.944	0.559	0.426	
	R3	0.220	7	1.062	1.898	
**BBP**	R1	0.009	0.074	0.021	0.018	1.22
	R2	0.00	0.197	0.039	0.049	
	R3	0.00	0.130	0.033	0.034	
**BEHP**	R1	1.032	185.432	17.267	46.698	4.35
	R2	0.368	326.211	24.24	83.564	
	R3	0.087	256	20.193	55.308	
**DOP**	R1	0.013	0.663	0.109	0.162	1.20
	R2	0.00	3.609	0.275	0.923	
	R3	0.002	55	3.848	14.161	
**Di- Octyl phthalate**	R1	0.347	1.189	0.570	0.232	–
	R2	0.037	0.250	0.088	0.056	
	R3	0.048	1.1	0.323	0.247	

R: sampling step.

In a study conducted by Karamianpour et al. on Accumulation, sources, and health risks of phthalic acid esters (PAEs) in road dust from heavily industrialized, urban and rural areas in southern Iran. They result proved the ƩPAEs concentration levels in the industrial areas was 37.8–82.5 μg/g. Also, they result showed most PAEs pollution researches focused on soil, sediment, or indoors but neglects street dust, which is also a significant pollution marker as humans, particularly in megacities, are at risk through direct/indirect exposure to street dusk. The amount of PEs reported in Asaluyeh and Bushehr in the south of Iran, on the northern side of the Persian Gulf was very similar to this study [29]. Another study conducted by Zhang et al. in the field of measuring phthalate esters in outdoor dust samples on Tibetan Plateau, China revealed that the PAEs concentrations between the 0.08–31.49 μg/g with a mean of 3.57 μg/g. This study demonstrated that High concentrations of PAEs in the outdoor dust in commercial districts, which were related to the heavy use of PAEs in the public commerce such as consumer products, commodities, and building materials [44].

**Fig. 2:**
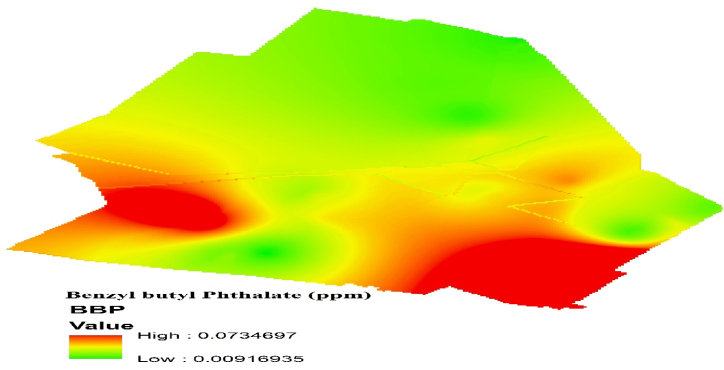
Spatial distribution of BBP concentration in the study area Using Geographical Information System.

Fig. 2. Depicts the zoning of BBP values utilizing Geographic Information System (GIS) mapping techniques. The spatial distribution of BBP values is visually represented through color-coded zones, indicating varying levels of potential health risks associated with exposure to phthalate esters (PAEs) across the study area. As can be seen in Fig. 2, the maximum BBP in the sample site of Yazd industrial park was higher than the other sample site, which is probably due to the textile industry, which has more than 5.4 million tons of synthetic fibers were produced worldwide, which can be the fabric washing process enters the environment in these areas.

### 3.2 Human health risk assessment and uncertainty analysis

#### 3.2.1 Deterministic approach

##### 3.2.1.1 Non-carcinogenic

The study focused on settled dust in the outdoor environment of the industrial park in Yazd. Fifteen sampling stations were selected across the outdoor environment of the industrial park for measuring the desired phthalate esters. The results depicted in Table 4, concerning the non-carcinogenic risk assessment with a focus on HQ associated with inhalation exposure, reveal varied potential health risks linked to different phthalates across various stations within the study area. It's notable that all HQ values are below one, indicating no non-carcinogenic risk associated with inhalation exposure. Since all HQs calculated from the inhalation exposure data are less than one, indicating values below the threshold for non-carcinogenic risk, it can be inferred that there is no non-carcinogenic risk associated with the inhalation exposure to phthalates in the study area [42].

**Table 4:** Inhalation exposure hazard quotients for phthalates in industrial dust samples.

Station	HQ Inhalation					
	DMP	DEP	DIBP	DnBP	BBP	DOP
1	1.6492 x10-^1^³	8.5315 x 10-^12^	1.11713 x 10-^10^	1.86881 x 10^−11^	4.00009 x 10-^12^	2.822 x 10^−11^
2	1.6157 x 10-^11^	1.8674 x 10^−11^	6.03694 x 10-^10^	1.20459 x 10^−11^	3.69809 x 10-^12^	7.55609 x 10^−11^
3	1.5256 x 10-^12^	7.7299 x 10-^12^	7.3091 x 10-^10^	1.57829 x 10^−11^	4.78795 x 10-^12^	4.63898 x 10^−11^
4	2.7593 x 10-^12^	6.23938 x 10-^12^	3.22217 x 10^−11^	1.38198 x 10^−11^	2.32351 x 10-^12^	3.05576 x 10^−11^
5	3.9363 x 10^−11^	3.33679 x 10^−11^	6.12623 x 10^−11^	1.15004 x 10^−11^	8.02267 x 10-^12^	2.339 x 10^−11^
6	3.9487 x10 ^−12^	7.71521 x 10-^12^	2.711 x 10^−9^	1.80244 x 10^−11^	1.51521 x 10^−11^	2.73453 x 10^−11^
7	7.1554 x10 ^−12^	6.61015 x 10-^12^	1.80545 x 10^−11^	4.34325 x 10-^12^	2.5506 x 10-^12^	1.5814 x 10^−11^
8	1.6492 x10 ^−13^	3.5183 x 10-^12^	4.27349 x 10^−11^	1.88885 x 10^−11^	2.17207 x 10-^12^	5.19642 x10 ^−12^
9	1.3523 x10 ^−12^	4.15459 x 10-^12^	3.48083 x 10^−11^	1.07282 x 10^−11^	1.91669 x 10-^12^	2.54239 x 10^−11^
10	1.6492 x10 ^−13^	7.09709 x 10-^12^	6.52585 x 10^−11^	1.27003 x 10^−11^	2.34719 x 10-^12^	3.0352 x 10^−11^
11	2.0438 x10 ^−12^	5.14317 x 10-^12^	3.10032 x 10^−11^	6.51806 x 10-^12^	8.81461 x 10-^12^	9.12178 x10 ^−12^
12	2.4635 x10 ^−12^	4.51354 x 10-^12^	3.2732 x 10^−11^	5.6444 x 10-^12^	3.48601 x 10-^12^	2.90882 x 10^−11^
13	1.6492 x10 ^−13^	3.94981 x 10-^12^	7.19513 x 10^−11^	2.59383 x 10^−11^	2.06383 x 10-^12^	4.11116 x 10^−11^
14	1.6492 x 10^−12^	5.12981 x 10-^12^	3.839 x 10^−11^	5.66187 x 10-^12^	2.84516 x 10-^12^	6.6471 x 10-^12^
15	1.1544 x10 ^−12^	3.73903x 10-^12^	1.29862 x 10^−10^	2.04426 x 10^−11^	1.88811 x 10-^12^	3.33427x 10^−11^
**Mean**	**4.947 x 10**^**−**^**^12^**	**8.245810–**^**12**^	**3.15402 x 10**^**−**^**^10^**	**3.09217 x 10**^**−**^**^11^**	**4.1229 x 10-**^**12**^	**4.5352 x 10**^**−**^**^11^**

Upon scrutinizing the data, it's evident that the HQ values for each phthalate vary across the stations, highlighting spatial differences in inhalation exposure risks within Yazd's industrial park. For instance, the HQ values for DMP range from 1.6492 x10 ^−13^ to 3.9487 x 10^−12^, while for DEP, they range from 3.5183 x 10-^12^to 8.5315x 10-^12^across different stations. Similarly, other phthalates such as DIBP, DnBP, BBP, and DOP exhibit variations in HQ values across the stations. While the spatial variability observed in the HQ values necessitates targeted mitigation strategies, the fact that all HQ values are below one indicates no non-carcinogenic risk associated with inhalation exposure to phthalates in the study area. Further analysis and interpretation of these results, along with other exposure pathways, will provide a comprehensive understanding of the overall non-carcinogenic health risks posed by phthalates in the industrial park.

Similarly, Table 5 presents the HQ values calculated for each phthalate across the fifteen sampling stations. The HQ values exhibit spatial variations, with different phthalates showing distinct ranges across the stations. For instance, DMP ranges from 1.6492 x 10^−13^ to 3.9487 x 10^−12^, while DEP varies from 3.5183 x 10^−12^ to 8.5315 x 10^−12^ across different locations. Similar variations are observed for other phthalates, including DIBP, DnBP, BBP, and DOP. Importantly, all HQ values in Table 4 are below one, indicating no non-carcinogenic risk associated with inhalation exposure to phthalates in the study area.

**Table 5:** Ingestion exposure hazard quotients for phthalates.

Stations	HQ Ingestion					
	DMP	DEP	DIBP	DnBP	BBP	DOP
1	7.1429 x 10-^10^	3.69517 x 10-^08^	7.5602 x 10-^07^	6.47538 x 10-^07^	6.9301 x 10-^07^	1.22229 x 10-^07^
2	6.9978 x 10-^08^	8.0881 x 10-^08^	4.08551 x 10-^06^	4.17387 x 10-^06^	6.40688 x 10-^07^	3.2727 x 10-^07^
3	6.6075 x 10-^09^	3.34798 x 10-^08^	4.94645 x 10-^06^	5.46871 x 10-^06^	8.29505 x 10-^08^	2.00924 x 10-^07^
4	1.1951 x 10-^08^	2.7024 x 10-^08^	2.18061 x 10-^07^	4.78852 x 10-^07^	4.02545 x 10-^08^	1.32351 x 10-^07^
5	1.7049- x 10-^07^	1.44523 x 10-^07^	4.14594 x 10-^07^	3.98485 x 10-^07^	1.38991 x 10-^07^	1.01307 x 10-^07^
6	1.7103 x 10-^08^	3.34162x 10-^08^	1.83467 x 10-^05^	6.24538 x 10-^07^	2.62507 x 10-^07^	1.18438 x 10-^06^
7	3.0991 x 10-^08^	2.86299 x 10-^08^	1.22184 x 10-^07^	1.50492 x 10-^07^	4.41888 x 10-^08^	6.84937 x 10-^08^
8	7.1429 x 10-^10^	1.52385 x 10-^08^	2.89209 x 10-^07^	6.54481 x 10-^07^	3.76307 x 10-^08^	2.25068 x 10-^08^
9	5.8571 x 10-^09^	1.79944 x 10-^08^	2.35565 x 10-^07^	3.7173 x 10-^07^	3.32064 x 10-^08^	1.10116 x 10-^07^
10	7.1429 x 10-^10^	3.0739 x 10-^08^	4.41638 x 10-^07^	4.40063 x 10-^07^	4.06648 x 10-^08^	1.31461 x 10-^07^
11	8.852 x 10-^09^	2.22761 x 10-^08^	2.09815 x 10-^07^	2.25849 x 10-^07^	1.52712 x 10-^07^	3.95083 x 10-^08^
12	1.067 x 10-^08^	1.95491 x 10-^08^	2.21514 x 10-^07^	1.95577 x 10-^07^	6.03945 x 10-^08^	1.25987 x 10-^07^
13	7.1429 x 10-^10^	1.71074 x 10-^08^	4.86932 x 10-^07^	8.98752 x 10-^07^	3.57555 x 10-^08^	1.78063 x 10-^07^
14	7.1429 x 10-^09^	2.22183 x 10-^08^	2.59805 x 10-^07^	1.96182 x 10-^07^	4.92919 x 10-^08^	2.879 x 10-^08^
15	5 x 10-^09^	1.61945 x 10-^08^	8.78844 x 10-^07^	7.08329 x 10-^07^	3.27112 x 10-^08^	1.44414 x 10-^07^
**Mean**	**2.143 x 10-**^**08**^	**3.57143 x 10-**^**08**^	**2.1345 x 10-**^**06**^	**1.07143** x **10-**^**06**^	**7.1429 x 10-**^**08**^	**1.96429 x 10-**^**07**^

Table 6 provides the HQ values calculated for each phthalate. This table highlights spatial variations in potential health risks associated with dermal exposure across different locations within the study area. Notably, all HQ values presented in Table 5 are below one, indicating that there is no non-carcinogenic risk associated with dermal exposure to phthalates in the industrial park of Yazd. Further analysis of these results, along with exploration of other exposure pathways, will contribute to a comprehensive understanding of the overall non-carcinogenic health risks posed by phthalates in the industrial park of Yazd.

**Table 6:** Dermal exposure hazard quotients for phthalates.

Stations	HQ dermal					
	DMP	DEP	DIBP	DnBP	BBP	DOP
1	4 x 10-^12^	2.0693 x 10-^10^	4.23371 x 10-^09^	3.62621 x 10-^09^	3.88085 x 10-^10^	6.8448E x 10-^10^
2	3.91877 x 10-^10^	4.52933 x 10-^10^	2.28789 x 10-^08^	2.33737 x 10-^08^	3.58785 x 10-^10^	1.83271 x 10-^09^
3	3.70021 x 10-^11^	1.87487 x 10-^10^	2.77001 x 10-^08^	3.06248 x 10-^08^	4.64523 x 10-^10^	1.12517 x 10-^09^
4	6.69269 x 10-^11^	1.51335 x 10-^10^	1.22114x 10-^09^	2.68157 x 10-^09^	2.25425 x 10-^10^	7.41167 x 10-^10^
5	9.54741 x 10-^10^	8.0933 x 10-^10^	2.32172 x 10-^09^	2.23152 x 10-^09^	7.78352 x 10-^10^	5.67319 x 10-^10^
6	9.5776 x 10-^11^	1.87131 x 10-^10^	1.02742x 10-^07^	3.49741x 10-^09^	1.47004 x 10-^09^	6.63253 x 10-^10^
7	1.73551 x 10-^10^	1.60328 x 10-^10^	6.84229x 10-^10^	8.42757 x 10-^10^	2.47457 x 10-^10^	3.83565 x 10-^10^
8	4 x 10-^12^	8.53357E x 10-^11^	1.61957x 10-^09^	3.66509 x 10-^09^	2.10732 x 10-^10^	1.26038 x 10-^10^
9	3.28 x 10-^11^	1.00769 x 10-^10^	1.31917 x 10-^09^	2.08169 x 10-^09^	1.85956 x 10-^10^	6.16651 x 10-^10^
10	4 x 10-^12^	1.72138 x 10-^10^	2.47317x 10-^09^	2.46435 x 10-^09^	2.27723 x 10-^10^	7.3618 x 10-^10^
11	4.95709 x 10-^11^	1.24746 x 10-^10^	1.17496 x 10-^09^	1.26475 x 10-^09^	8.55185 x 10-^10^	2.21247 x 10-^10^
12	5.9752 x 10-^11^	1.09475 x 10-^10^	1.24048 x 10-^09^	1.09523 x 10-^09^	3.38209 x 10-^10^	7.05527 x 10-^10^
13	4 x 10-^12^	9.58017 x 10-^11^	2.72682 x 10-^09^	5.03301 x 10-^09^	2.00231 x 10-^10^	9.97153 x 10-^10^
14	4 x 10-^11^	1.24422 x 10-^10^	1.45491 x 10-^09^	1.09862 x 10-^09^	2.76035 x 10-^10^	1.61224 x 10-^10^
15	2.8 x 10-^11^	9.06893 x 10-^11^	4.92153 x 10-^09^	3.96664 x 10-^09^	1.83183 x 10-^10^	8.0872 x 10-^10^
**Mean**	**1.2 x 10-**^**10**^	**2 x 10-**^**10**^	**1.19531** x **10-**^**08**^	**6** x **10-**^**8**^	**4** x **10-**^**10**^	**1.1** x **10-**^**09**^

Table 7 presents the computed HI values for each phthalate across the fifteen sampling sites. The HI serves as an aggregate indicator of the non-carcinogenic health risks linked to exposure to various contaminants. In this study, the HI values were determined by aggregating the individual HQs derived from inhalation, ingestion, and dermal exposure pathways, as detailed in Table 4, Table 5, Table 6.

**Table 7:** Hazard index (HI) for phthalates in industrial dust samples.

Station	HI					
	DMP	DEP	DIBP	DnBP	BBP	DOP
1	7.18451 x 10-^10^	3.71672 x 10-^08^	7.60366 x 10-^07^	6.51183 x 10-^07^	6.9693 x 10-^08^	1.22941 x 10-^07^
2	7.03861 x 10-^08^	8.13526 x 10-^08^	4.10899 x 10-^06^	4.19737 x 10-^06^	6.44313 x 10-^08^	3.29179 x 10-^07^
3	6.64605 x 10-^09^	3.3675 x 10-^08^	4.97488 x 10-^06^	5.4995 x 10-^06^	8.34198 x 10-^08^	2.02095 x 10-^07^
4	1.20209 x 10-^08^	2.71816 x 10-^08^	2.19314 x 10-^07^	4.81548 x 10-^07^	4.04823 x 10-^08^	1.33123 x 10-^07^
5	1.71484 x 10-^07^	1.45366 x 10-^07^	4.16977 x 10-^07^	4.00728 x 10-^07^	1.39778 x 10-^07^	1.01898 x 10-^07^
6	1.72026 x 10-^08^	3.3611 x 10-^08^	1.84522 x 10-^05^	6.28054 x 10-^07^	2.63992 x 10-^07^	1.19129 x 10-^06^
7	3.1172 x 10-^08^	2.87969 x 10-^08^	1.22886 x 10-^07^	1.51339 x 10-^07^	4.44388 x 10-^08^	6.88931 x 10-^08^
8	7.18451 x 10-^10^	1.53274 x 10-^08^	2.90872 x 10-^07^	6.58165 x 10-^07^	3.78436 x 10-^08^	2.2638 x 10-^08^
9	5.8913 x 10-^09^	1.80993 x 10-^08^	2.36919 x 10-^07^	3.73822 x 10-^07^	3.33943 x 10-^08^	1.10758 x 10-^07^
10	7.18451E x 10-^10^	3.09182 x 10-^08^	4.44176 x 10-^07^	4.4254 x 10-^07^	4.08948 x 10-^08^	1.32227 x 10-^07^
11	8.90357 x 10-^09^	2.2406 x 10-^08^	2.11021 x 10-^07^	2.2712 x 10-^07^	1.53576 x 10-^07^	3.97387 x 10-^08^
12	1.07322 x 10-^08^	1.96631 x 10-^08^	2.22787 x 10-^07^	1.96678 x 10-^07^	6.07362 x 10-^08^	1.26722 x 10-^07^
13	7.18451 x 10-^10^	1.72072 x 10-^08^	4.89731 x 10-^07^	9.03811 x 10-^07^	3.59578 x 10-^08^	1.79101 x 10-^07^
14	7.18451 x 10-^09^	2.23478 x 10-^08^	2.61298 x 10-^07^	1.97286 x 10-^07^	4.95708 x 10-^08^	2.89579 x 10-^08^
15	5.02915 x 10-^09^	1.6289 x 10-^08^	8.83895 x 10-^07^	7.12316 x 10-^07^	3.28963 x 10-^08^	1.45256 x 10-^07^
**Mean**	**2.1554 x 10-**^**08**^	**3.59225 x 10-**^**08**^	**2.14676 x 10-**^**06**^	**1.07746 x 10-**^**06**^	**7.1833 x 10-**^**08**^	**1.97574 x 10-**^**07**^

The observed spatial variation in HI values emphasizes the need to account for multiple exposure pathways when evaluating overall health risks related to environmental contaminants. It is also important to highlight that all HI values listed in Table 7 are below one, demonstrating that the total non-carcinogenic health risk from exposure to phthalates through inhalation, ingestion, and dermal pathways is within acceptable levels in the industrial park in Yazd. This indicates that, despite the differences in individual HQ values across different sampling stations, the overall health risk remains minimal and does not pose a significant threat to human health.

In a study conducted by Zhang et al. on-health risk assessment of phthalate esters in outdoor dust samples on Tibetan Plateau, China. They result proved total intakes of PAEs from outdoor dusts for children and adults were 1.50 × 10^−5^ and 2.47 × 10^−6^ mg·kg−1 d−1, respectively. Also, this research showed health risks assessment of the six PAEs via outdoor dust are currently acceptable. Therefore, estimated health risks of the six PAEs via outdoor dust are currently acceptable [44]. So, the results of this study are in line with the results of our research.

#### 3.2.2 Carcinogenic deterministic approach

The carcinogenic risk assessment in this study focuses specifically on the compound BBP. It is important to note that this assessment is tailored to the adult occupational population in the Industrial Park of Yazd, specifically focusing on industrial activities. Children are excluded from the exposure assessment given the study's occupational focus, with their exposure assumed to be nonexistent. This method ensures a focused evaluation of CR linked with BBP exposure among the adult workforce in the industrial environment. The results presented in Table 8 demonstrate the CR calculated for BBP through different exposure pathways: inhalation (CR_inh_), ingestion (CR_ing_), and dermal exposure (CR_der_).

**Table 8:** Carcinogenic risk assessment of benzyl butyl phthalate (BBP).

Station	CR _inh_	CR _ing_	CR _der_
1	4.99737 x 10-^15^	2.16447 x 10-^11^	1.2121 x 10-^13^
2	4.62008 x 10-^15^	2.00105 x 10-^11^	1.12059 x 10-^13^
3	5.98166 x 10-^15^	2.59078 x 10-^11^	1.45084 x 10-^13^
4	2.9028 x 10-^15^	1.25726 x 10-^11^	7.04068 x 10-^14^
5	1.00228 x 10-^14^	4.3411 x 10-^11^	2.43102 x 10-^13^
6	1.89297 x 10-^14^	8.19885 x 10-^11^	4.59136 x 10-^13^
7	3.18651 x 10-^15^	1.38014 x 10-^11^	7.7288 x 10-^14^
8	2.7136 x 10-^15^	1.17532 x 10-^11^	6.58177 x 10-^14^
9	2.39456 x 10-^15^	1.03713 x 10-^11^	5.80794 x 10-^14^
10	2.93238 x 10-^15^	1.27008 x 10-^11^	7.11243 x 10-^14^
11	1.10122 x 10-^14^	4.76962 x 10-^11^	2.67099 x 10-^13^
12	4.35512 x 10-^15^	1.88629 x 10-^11^	1.05633 x 10-^13^
13	2.57837 x 10-^15^	1.11675 x 10-^11^	6.25378 x 10-^14^
14	3.5545 x 10-^15^	1.53953 x 10-^11^	8.62136 x 10-^14^
15	2.35884 x 10-^15^	1.02166 x 10-^11^	5.72132 x 10-^14^
**Mean**	**5.5027** x 10-^15^	**2.38334** x 10-^11^	**1.33467** x 10-^13^

Upon analysis of the CR values, it is evident that BBP poses a minimal carcinogenic risk through all exposure pathways. The calculated CR values for BBP across all exposure routes are consistently low, with magnitudes ranging from 10× 10-^15^ to 10 x 10-^11^. The cancer risk from deposited in the outdoor environment of Yazd industrial Park was found to be in the order: ingestion > dermal > inhalation These values indicate extremely low cancer risks associated with BBP exposure in the studied industrial setting. When the CR values exceed 10E-6, it suggests that the exposure of individuals to PAEs could potentially lead to carcinogenic risks. In other words, CR values greater than this threshold indicate a heightened level of concern regarding the potential induction of cancer due to PAE exposure. The low CR values suggest that the occupational exposure to BBP in the Industrial Park of Yazd poses negligible carcinogenic risks to the adult workforce. However, it is essential to continue monitoring and implementing preventive measures to minimize any potential health risks associated with BBP exposure, ensuring the safety and well-being of the industrial workers [44].

#### 3.2.3 Non-carcinogenic probabilistic approach

The probabilistic approach is utilized to assess the non-carcinogenic and carcinogenic health risks associated with exposure to six phthalates in dust samples collected from the Industrial Park of Yazd City. The assessment is conducted across three exposure pathways: inhalation, ingestion, and dermal. The results of the probabilistic assessment provide valuable insights into the range of potential health risks posed by phthalate exposure in the industrial environment, facilitating informed decision-making for public health interventions and environmental management strategies. In this study, the SA was used to determine which pathways and variables effect the risk estimate. SA determines how the input factors have a proper effect on the uncertainty of the final response [43].

##### 3.2.3.1 Inhalation exposure

**Fig. 3:**
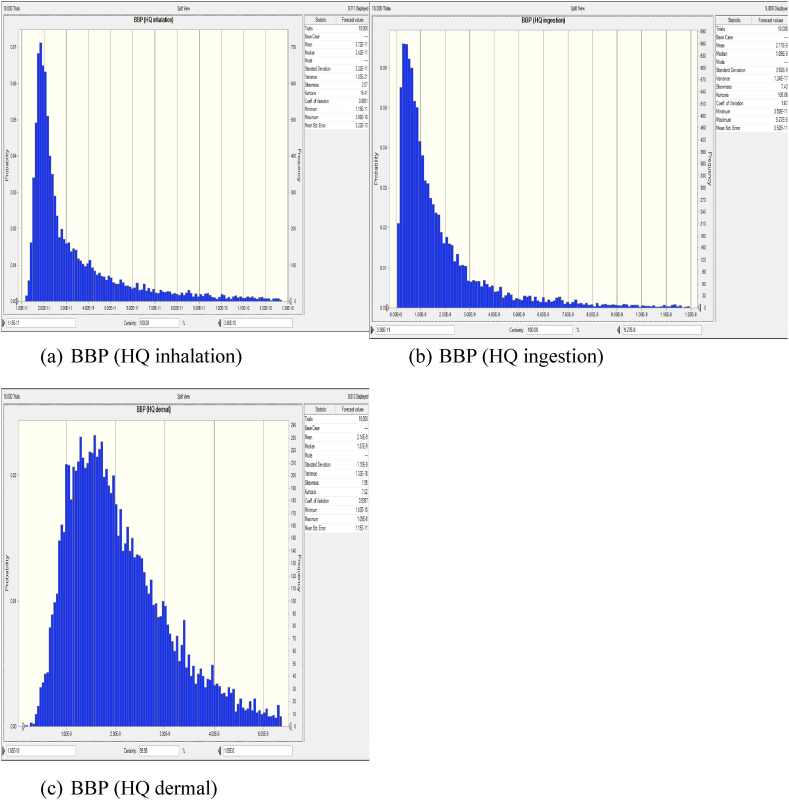
Probability Distribution of BBP inhalation(a), ingestion(b), and dermal(c) using Monte Carlo Simulation in Crystal Ball. (a) BBP sensitivity (HQ inhalation) (b) BBP sensitivity (HQ ingestion) (c) BBP sensitivity (HQ dermal).

The variability in inhalation exposure and resulting health risks are comprehensively analyzed using Monte Carlo simulation techniques and probability distributions for key parameters such as inhalation rate, exposure frequency, and phthalate concentration. Fig. 3 illustrates a frequency chart generated through Monte Carlo simulation, depicting the distribution of HQ values specifically for BBP.

This chart illustrates the probability distribution of Hazard Quotient (HQ) values, facilitating a probabilistic evaluation of the potential health risks associated with BBP inhalation exposure. As shown in Fig. 3(a), the Monte Carlo simulation results reveal variability in HQ values across different percentile ranges. Specifically, the HQ values for BBP range from a minimum of 1.15 × 10⁻^11^ to a maximum of 3.86 × 10⁻^1^⁰. These extremely low HQ values suggest that the health risk associated with BBP inhalation for the studied population is negligible, as HQ values below 1 indicate no significant non-carcinogenic risk. The distribution of HQ values further underscores the consistency of low-risk levels across different exposure scenarios, reinforcing the conclusion that BBP inhalation does not pose a significant health threat in this industrial.

While the initial estimation of inhalation exposure to BBP at the regional background site was below the U.S. EPA's standard, individuals in urban and suburban areas experienced higher exposure due to various emission sources. Supplementary Fig. S2 provide frequency charts depicting the probabilistic assessment results for five other phthalates investigated in this study, namely DMP, DEP, DIBP, DnBP, and DOP. These figures offer a comprehensive overview of the distribution of HQ values across different exposure scenarios and percentiles. Similar to Fig. 3, all supplementary figures demonstrate that the calculated HQ values for these phthalates remain below one, indicating no non-carcinogenic risk associated with exposure to these compounds in the studied environment (Fig. S3).

**Fig. 4:**
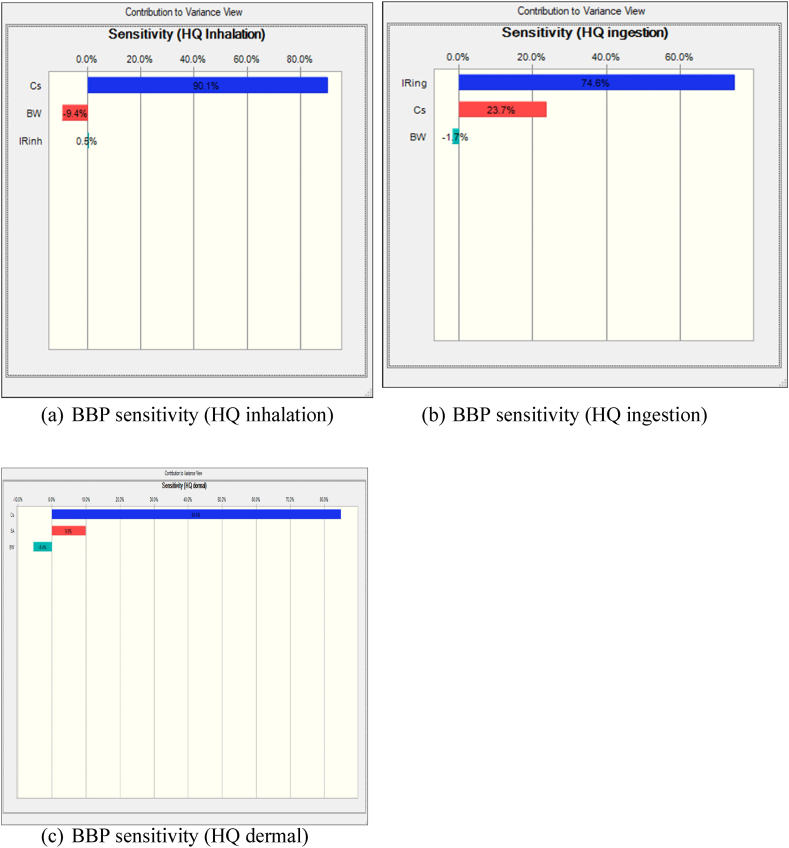
Sensitivity Analysis of BBP inhalation(a), ingestion(b), and dermal(c) Using Monte Carlo Simulation in Crystal Ball.

Also, sensitivity analysis, a crucial component of risk assessment methodologies, was conducted using Monte Carlo simulation in Crystal Ball to elucidate the influential variables affecting health risks associated with exposure to BBP through inhalation. This method systematically explores the variability of input parameters to assess their impact on the uncertainty of the final response, providing insights into the most significant contributors to health risk within the exposed population. Fig. 4 (a) illustrates the results of sensitivity analysis conducted on BBP exposure through inhalation using Monte Carlo simulation in Crystal Ball. According to this figure, BBP concentration exhibited the most substantial influence on increasing the non-carcinogenic risk for the exposed population groups. Consequently, a reduction in BBP concentration could effectively mitigate health risks associated with exposure to this compound. Furthermore, the sensitivity analysis identified BW as another influential factor inversely related to sensitivity, indicating its significant impact on risk assessment following BBP concentration. Supplementary Fig. S3 provide sensitivity analysis results for five other phthalates investigated in this study, namely DMP, DEP, DIBP, DnBP, and DOP.

##### 3.2.3.2 Ingestion exposure

The non-carcinogenic risks associated with ingestion exposure to six phthalates found in dust samples from the Industrial Park of Yazd city were investigated using a probabilistic approach. We analyze the variability in ingestion exposure by considering factors such as ingestion rate, exposure frequency, and phthalate concentration. This assessment provides critical insights into the potential health hazards posed by ingesting phthalates in the industrial environment, contributing to informed risk management decisions and protective measures for public health. The results presented in Fig. 3(b) show that the Hazard Quotient (HQ) values for the adult occupational population vary between 3.05 × 10⁻^11^and 9.02 × 10⁻^8^. This average HQ is well below the threshold of 1, indicating that the non-carcinogenic risk associated with BBP exposure for the adult workforce is minimal. Additionally, the sensitivity analysis depicted in Fig. 4(b) highlights that the Ingestion Rate has the most significant positive effect on the HQ compared to other factors, such as BBP concentration and Body Weight. This finding underscores the importance of ingestion as a key determinant of overall health risk, suggesting that measures aimed at reducing ingestion exposure could further lower the already minimal health risk in this occupational setting.

##### 3.2.3.3 Dermal exposure

This section examines the non-carcinogenic risks linked to dermal exposure to phthalates, using a probabilistic approach to account for variability in dermal exposure. Key parameters such as skin adherence, exposure duration, and phthalate concentration were considered. According to the results shown in Fig. 3(c), the probability HQ values for the adult occupational population ranged from 1.6 × 10⁻^10^to 1.09 × 10⁻^8^. These values indicate that the risk of non-carcinogenic effects from dermal exposure is very low. Furthermore, the sensitivity analysis presented in Fig. 4(c) revealed that BBP concentration (C) has the largest positive impact on the HQ compared to other input variables, such as Skin Adherence Rate (SR) and Body Weight (BW). This suggests that BBP concentration is the most critical factor in determining the overall health risk, highlighting the importance of controlling phthalate concentrations to further minimize the already low risk of non-carcinogenic effects.

#### 3.2.4 Carcinogenic probabilistic approach

phthalate esters include DMP, DEP, DnBP, DEHP, BBP and DnOP are classified as a pollutants Priority and dangerous according EPA category, therefore they can potentially enhance the risk of cancer in humans. Long term exposure to low amounts of toxic metals could, therefore, result in many types of cancers. The BBP investigated in the study have the potential to elevate the risk of cancer in humans [36]. The analysis of CR probabilities for Inhalation, Ingestion and Dermal exposure measurements in adult workforce in the industrial setting reveals some patterns. The cancer risk from deposited in the outdoor environment of Yazd industrial Park was found to be in the order: dermal ingestion > dermal > inhalation.

##### 3.2.4.1 Inhalation exposure

**Fig. 5:**
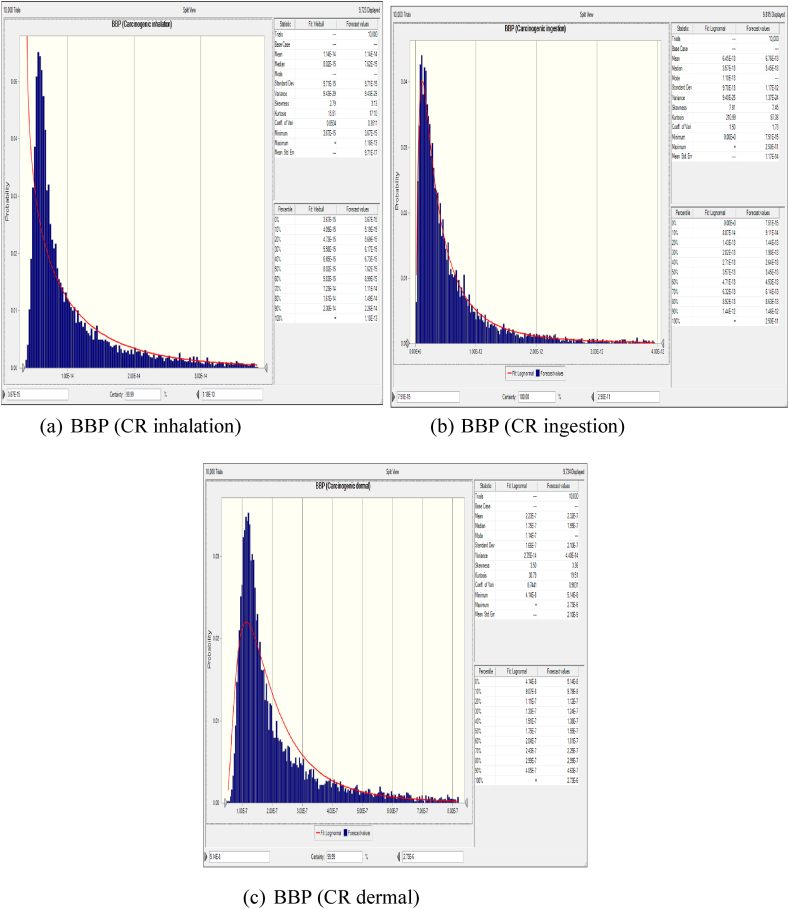
Probability Distribution of BBP inhalation(a), ingestion(b), and dermal(c) Cancer Risk.

**Fig. 6:**
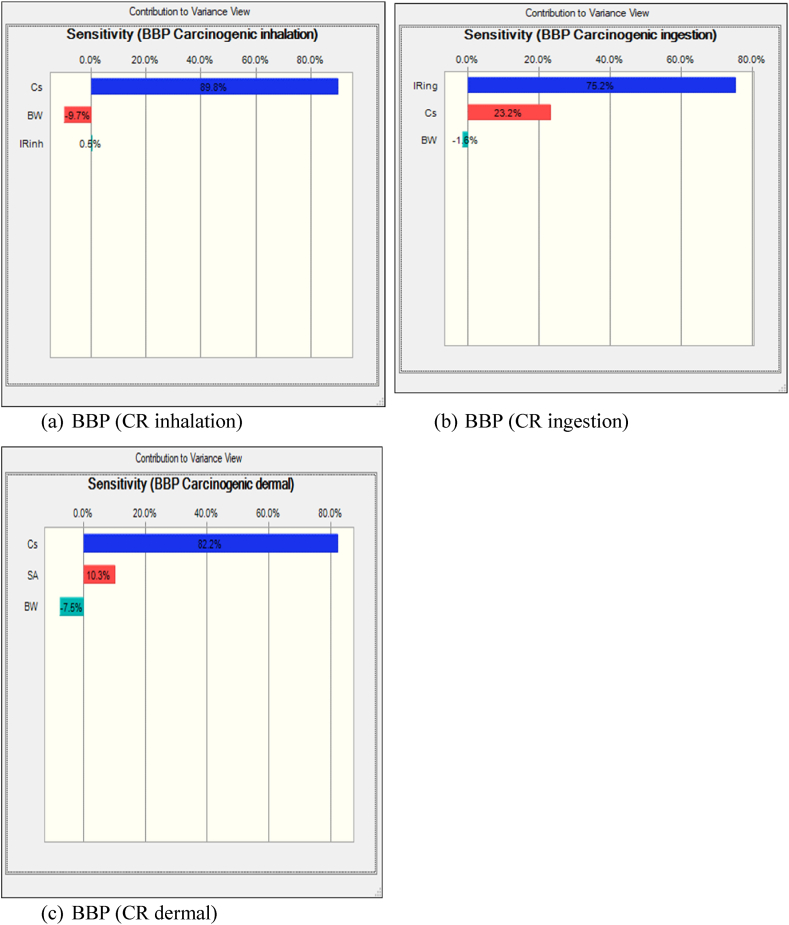
Probability Distribution of BBP inhalation(a), ingestion(b), and dermal(c) Cancer Risk.

Fig. 5 presents a frequency chart generated through Monte Carlo simulation, depicting the distribution of CR probability values specifically for BBP. This figure provides insights into the probability distribution of CR values, allowing for a probabilistic assessment of the potential health risks associated with BBP inhalation exposure. According to the Monte Carlo simulation results depicted in Fig. 5(a), the CR values for BBP exhibit variability across different percentile ranges. Specifically, the 5th percentile (P5) corresponds to a CR value of 4.84 × 10⁻^15^, indicating that 5 % of the simulated outcomes resulted in CR values equal to or lower than this threshold. Conversely, the 95th percentile (P95) corresponds to a CR value of 3.09 × 10⁻^14^, suggesting that 95 % of the simulated outcomes yielded CR values equal to or lower than this value. According to Fig. 6(a), BBP concentration exhibited the most influence on increasing the carcinogenic risk for the exposed population groups. Consequently, reducing BBP concentration could effectively mitigate health risks associated with exposure to this compound. Furthermore, the sensitivity analysis identified BW as another influential factor inversely related to sensitivity, indicating its significant impact on risk assessment following BBP concentration.

##### 3.2.4.2 Ingestion exposure

From the results shown in Fig. 5 (b), the CR value associated with BBP ingestion dust deposited in the outdoor environment for adult occupational population ranged from 7.5 x 10-^15^ to 2.5 x 10-^11^, with a mean of 6.76 x 10-^13^. In addition, the results of the sensitivity analysis Fig. 6 (b) showed that Ingestion rate (IR) had the largest positive impact on CR compared to other input variables (e.g., Concentration and Body Weight.

##### 3.2.4.3 Dermal exposure

From the results shown in Fig. 5 (c), the CR value for infants ranged from 5.14 x 10-^8^ to 2.73 x 10-^6^, with a mean of 2.32 x 10-^7^. The analysis of CR due to dermal contact in adult's work place reveals that this group consistently have lower CR values for all parameters. Fig. 6 (c) indicate the sensitivity analysis of variables in computing CR for adult occupational population groups. According to Fig. 6 c, the most influential parameter in the carcinogenic risk in adult occupational population groups is Ingestion rate, Concentration and Body Weight. The study conducted by Li et al., on human risk assessment to exposure PAEs in road dust sample, sensitivity analysis results proved that concentrations, ingestion rate and fraction of PAEs absorbed in the skin were most important parameters on the assessment of exposure risk of PAEs via street dust [28].

## 4 Study limitations and ideas for future researches

In this study, due to the high costs of the device analysis, only the concentration of 8 types of PAES has been investigated. Meanwhile, other metabolites were ignored. Therefore, it is recommended to investigate more metabolites in future studies.

Suggestions for future studies.

1 humans spent over 80 % of their time in indoor environments in their daily lives, and indoor environment quality had a significant impact on human health, it should also be measured in the staff office room of Yazd Industrial City.
2 Sampling should be done in different periods of time in different seasons on cloudy, sunny, rainy, stormy days.
3 Health risk assessment should be done using biomarkers.
4 The concentration of phthalate esters in the wastewater of the industrial town and water should be measured.

## 5 Conclusions

In this study, the PEs accumulated in in dust deposited in the outdoor environment were investigated in Yazd industrial Park. Among the eight commonly-phthalate investigated, the highest and lowest are related to BEHP and DMP in stations S8 and S6. The total HI values for adult occupational population group were below one in dust deposited in the outdoor environment of all sampling station. In generally, indicating that the combined non-carcinogenic health risk from exposure to phthalates via inhalation, ingestion, and dermal pathways is within acceptable levels in the industrial park in Yazd. Therefore, the probabilistic risk estimation method using Monte Carlo simulation showed that PEs hardly posed non-carcinogenic or cancer risks to adult. This suggests that, despite variations in individual HQ values across different sampling stations, the cumulative health risk remains low and does not pose a significant threat to human health.

This study provides valuable information on PEs concentrations in dust deposited in the outdoor environment, and their daily intakes through different routes, which can help to support the prevention and risk control of PEs pollution in different age grope and exposure scenarios.

## Funding

This study was conducted with the financial support of 10.13039/501100015034Shahid Sadoughi University of Medical Sciences, Yazd, Iran by grant number SSU.SPH.REC.1401.056″ title = "http://ethics.research.ac.ir/IR.SSU.SPH.REC.1401.056">1401.056.

## Ethical approval

All authors have read, understood, and have complied as applicable with the statement on “Ethical responsibilities of Authors” as found in the Instructions for Authors.

## Ethical approval

Approved No. of ethic committee: IR.SSU.SPH.REC.1401.056.

## Declarations

The datasets used and/or analyzed during the current study are fully presented in the paper. Should any raw data files be needed in another format they are available from the corresponding author upon reasonable request.

## Data availability

Data will be made available on request.

## CRediT authorship contribution statement

**Mohammad Hasan Ehrampush:** Writing – original draft, Supervision, Conceptualization. **Ehsan Abouee:** Writing – original draft, Software, Methodology, Formal analysis. **Hossein Arfaeinia:** Writing – original draft, Validation, Methodology. **Zahra soltanian:** Writing – original draft, Software, Investigation. **Mahdi Ghorbanian:** Writing – original draft, Investigation. **Sahar Ghalehaskari:** Writing – original draft, Software, Methodology, Investigation.

## Declaration of competing interest

The authors declare that they have no known competing financial interests or personal relationships that could have appeared to influence the work reported in this paper.

## References

[1] Liu F.-F., Liu G.-z., Zhu Z.-l., Wang S.-c., Zhao F.-f. (2019). Interactions between microplastics and phthalate esters as affected by microplastics characteristics and solution chemistry. Chemosphere.

[2] Walker T.R., Fequet L. (2023). Current trends of unsustainable plastic production and micro (nano) plastic pollution. TrAC, Trends Anal. Chem..

[3] Puri M., Gandhi K., Kumar M.S. (2023). The occurrence, fate, toxicity, and biodegradation of phthalate esters: an overview. Water Environ. Res..

[4] Asrin N.R.N., Dipareza A. (2019). Microplastics in ambient air (case study: Urip sumoharjo street and mayjend sungkono street of surabaya city, Indonesia). IAETSD J. Adv. Res. Appl. Sci.

[5] Baloyi N., Tekere M., Maphangwa K., Masindi V. (2021). Insights into the prevalence and impacts of phthalate esters in aquatic ecosystems. Front. Environ. Sci..

[6] Bowley J., Baker-Austin C., Porter A., Hartnell R., Lewis C. (2021). Oceanic hitchhikers–assessing pathogen risks from marine microplastic. Trends Microbiol..

[7] Okoye C.O., Addey C.I., Oderinde O., Okoro J.O., Uwamungu J.Y., Ikechukwu C.K., Okeke E.S., Ejeromedoghene O., Odii E.C. (2022). Toxic chemicals and persistent organic pollutants associated with micro-and nanoplastics pollution. Chemical Engineering Journal Advances.

[8] Verster C., Minnaar K., Bouwman H. (2017). Marine and freshwater microplastic research in South Africa. Integrated Environ. Assess. Manag..

[9] Li X., Zhang W., Lv J., Liu W., Sun S., Guo C., Xu J. (2021). Distribution, source apportionment, and health risk assessment of phthalate esters in indoor dust samples across China. Environ. Sci. Eur..

[10] McKee R.H., Butala J.H., David R.M., Gans G. (2004). NTP center for the evaluation of risks to human reproduction reports on phthalates: addressing the data gaps. Reprod. Toxicol..

[11] Scholz N. (2003). Ecotoxicity and biodegradation of phthalate monoesters. Chemosphere.

[12] Wang X., Hu E., Yang C., Li M. (2023). Occurrence, distribution and risk assessment of phthalate esters in 51 urban wastewater treatment plants in Shaanxi Province, China. J. Environ. Chem. Eng..

[13] Fernández I., Ruiz M. (2009). Descriptive model and evaluation system to locate sustainable industrial areas. J. Clean. Prod..

[14] Rostami R., Moussavi G., Jafari A.J., Darbari S. (2020). A modeling concept on removal of VOCs in wire-tube non-thermal plasma, considering electrical and structural factors. Environ. Monit. Assess..

[15] Sun K., Song Y., He F., Jing M., Tang J., Liu R. (2021). A review of human and animals exposure to polycyclic aromatic hydrocarbons: health risk and adverse effects, photo-induced toxicity and regulating effect of microplastics. Sci. Total Environ..

[16] Wang G., Lu J., Li W., Ning J., Zhou L., Tong Y., Liu Z., Zhou H., Xiayihazi N. (2021). Seasonal variation and risk assessment of microplastics in surface water of the Manas River Basin, China. Ecotoxicology and environmental safety.

[17] He Y., Wang Q., He W., Xu F. (2019). Phthalate esters (PAEs) in atmospheric particles around a large shallow natural lake (Lake Chaohu, China). Science of the total environment.

[18] Hei W., Li X., Gao G., Wang S., Zhang R., Wang K. (2022). Air pollutants and CO2 emissions in industrial parks and evaluation of their green upgrade on regional air quality improvement: a case study of seven cities in Henan Province. Atmosphere.

[19] Kim U.-J., Wang Y., Li W., Kannan K. (2019). Occurrence of and human exposure to organophosphate flame retardants/plasticizers in indoor air and dust from various microenvironments in the United States. Environ. Int..

[20] Lee Y.-M., Lee J.-E., Choe W., Kim T., Lee J.-Y., Kho Y., Choi K., Zoh K.-D. (2019). Distribution of phthalate esters in air, water, sediments, and fish in the Asan Lake of Korea. Environ. Int..

[21] Patchaiyappan A., Dowarah K., Ahmed S.Z., Prabakaran M., Jayakumar S., Thirunavukkarasu C., Devipriya S.P. (2021). Prevalence and characteristics of microplastics present in the street dust collected from Chennai metropolitan city, India. Chemosphere.

[22] Razeghi N., Hamidian A.H., Wu C., Zhang Y., Yang M. (2021). Scientific studies on microplastics pollution in Iran: an in-depth review of the published articles. Mar. Pollut. Bull..

[23] Sun C., Chen L., Zhao S., Guo W., Luo Y., Wang L., Tang L., Li F., Zhang J. (2021). Seasonal distribution and ecological risk of phthalate esters in surface water and marine organisms of the Bohai Sea. Mar. Pollut. Bull..

[24] Zhang Z.-M., Zhang H.-H., Zhang J., Wang Q.-W., Yang G.-P. (2018). Occurrence, distribution, and ecological risks of phthalate esters in the seawater and sediment of Changjiang River Estuary and its adjacent area. Sci. Total Environ..

[25] Zhao X., Shen J.-m., Zhang H., Li X., Chen Z.-l., Wang X.-c. (2020). The occurrence and spatial distribution of phthalate esters (PAEs) in the Lanzhou section of the Yellow River. Environ. Sci. Pollut. Control Ser..

[26] Zhu Q., Xu L., Wang W., Liu W., Liao C., Jiang G. (2022). Occurrence, spatial distribution and ecological risk assessment of phthalate esters in water, soil and sediment from Yangtze River Delta, China. Sci. Total Environ..

[27] Hou S., Zhao X., Liu Y., Tillotson M.R., Weng S., Wang H., Li Y., Liu B., Feng K., Zhang N. (2022). Spatial analysis connects excess water pollution discharge, industrial production, and consumption at the sectoral level. Npj Clean Water.

[28] Li B., Zhao Z.-B., Thapa S., Sun S.-J., Ma L.-X., Geng J.-L., Wang K., Qi H. (2020). Occurrence, distribution and human exposure of phthalic esters in road dust samples across China. Environ. Res..

[29] Karamianpour J., Arfaeinia H., Vakilabadi D.R., Ramavandi B., Dobaradaran S., Fazlzadeh M., Torkshavand Z., Banafshehafshan S., Shekarizadeh H., Ahmadi S. (2023). Accumulation, sources, and health risks of phthalic acid esters (PAEs) in road dust from heavily industrialized, urban and rural areas in southern Iran. Heliyon.

[30] Kong S., Ji Y., Liu L., Chen L., Zhao X., Wang J., Bai Z., Sun Z. (2012). Diversities of phthalate esters in suburban agricultural soils and wasteland soil appeared with urbanization in China. Environmental Pollution.

[31] Wang J., Yuan F., Ye H., Bu Z. (2023). Measurement of phthalates in settled dust in university dormitories and its implications for exposure assessment. Atmosphere.

[32] Wang L., Zhang W., Tao W., Wang L., Shi X., Lu X. (2017). Investigating into composition, distribution, sources and health risk of phthalic acid esters in street dust of Xi’an City, Northwest China. Environ. Geochem. Health.

[33] Xia M., Ouyang X., Wang X., Shen X., Zhan Y. (2018). Occupational exposure assessment of phthalate esters in indoor and outdoor microenvironments. Journal of Environmental Sciences.

[34] Abdi S., Sobhanardakani S., Lorestani B., Cheraghi M., Panahi H.A. (2021). Analysis and health risk assessment of phthalate esters (PAEs) in indoor dust of preschool and elementary school centers in city of Tehran, Iran. Environ. Sci. Pollut. Control Ser..

[35] Wang L., Liu M., Tao W., Zhang W., Wang L., Shi X., Lu X., Li X. (2018). Pollution characteristics and health risk assessment of phthalate esters in urban soil in the typical semi-arid city of Xi'an, Northwest China. Chemosphere.

[36] Wu L., Li X., Fan J., Bai Y., Zhang Y., Lu H., Guo C., Xu J. (2023). Distribution characteristics, source attribution, and health risk assessment of organophosphate esters in indoor and outdoor dust from various microenvironments in Beijing. Ecotoxicol. Environ. Saf..

[37] Wang J., Chen G., Christie P., Zhang M., Luo Y., Teng Y. (2015). Occurrence and risk assessment of phthalate esters (PAEs) in vegetables and soils of suburban plastic film greenhouses. Sci. Total Environ..

[38] Dehghani M.H., Zarei A., Yousefi M., Asghari F.B., Haghighat G.A. (2019). Fluoride contamination in groundwater resources in the southern Iran and its related human health risks. Desalination Water Treat..

[39] Gao K., Wang L., Xu Y., Zhang Y., Li H., Fu J., Fu J., Lu L., Qiu X., Zhu T. (2024). Concentration identification and endpoint-oriented health risk assessments on a broad-spectrum of organic compounds in atmospheric fine particles: a sampling experimental study in Beijing, China. Sci. Total Environ..

[40] Qasemi M., Ghorbani M., Salehi R., Attari S.M., Afsharnia M., Dehghani M.H., Farhang M., Zarei A., Gholinejad A., Zarei A. (2024). Human health risk associated with nitrates in some vegetables: a case study in Gonabad. Food Chemistry Advances.

[41] Zhu H., Zheng N., Chen C., Li N., An Q., Zhang W., Lin Q., Xiu Z., Sun S., Li X., Li Y., Wang S. (2024). Multi-source exposure and health risks of phthalates among university students in Northeastern China. Sci. Total Environ..

[42] Miao Y., Wang R., Lu C., Zhao J., Deng Q. (2017). Lifetime cancer risk assessment for inhalation exposure to di(2-ethylhexyl) phthalate (DEHP). Environ. Sci. Pollut. Control Ser..

[43] Jeong S.-H., Jang J.-H., Cho H.-Y., Lee Y.-B. (2021). Human risk assessment of di-isobutyl phthalate through the application of a developed physiologically based pharmacokinetic model of di-isobutyl phthalate and its major metabolite mono-isobutyl phthalate. Arch. Toxicol..

[44] Zhang Y., Li X., Zhang H., Liu W., Liu Y., Guo C., Xu J., Wu F. (2022). Distribution, source apportionment and health risk assessment of phthalate esters in outdoor dust samples on Tibetan Plateau, China. Sci. Total Environ..

