# Supplementary material for: Occurrence, distribution and risk assessment of phthalate esters in dust deposited in the outdoor environment of Yazd industrial park using Monte Carlo simulation
Source: Heliyon. 2024 Sep 8;10(18):e37500. doi: 10.1016/j.heliyon.2024.e37500 (PMC11416271; doi:10.1016/j.heliyon.2024.e37500)
Supplement: Multimedia component 1 [file mmc1.docx]

**Table S1. LOD and LOQ**

| **PEs** | **DLRa (ng mL−1)** | **R2** | **LODb (ng mL−1)** | **LOQc (ng mL−1)** |
| --- | --- | --- | --- | --- |
| **DMP** | 5-1000 | 0.993 | 1.68 | 5.56 |
| **DEP** | 5-1000 | 0.995 | 0.86 | 2.84 |
| **DBP** | 5-1000 | 0.990 | 0.75 | 2.46 |
| **BEHP** | 5-1000 | 0.991 | 0.10 | 0.34 |
| **BBP** | 5-1000 | 0.992 | 1.67 | 5.50 |
| **DNOP** | 5-1000 | 0.996 | 2.11 | 6.97 |

**Table S2. Relative standard deviation**

|  |  |  |  |  |  |  |  | **intraday** |
| --- | --- | --- | --- | --- | --- | --- | --- | --- |
| **No** | **Compound Name** | **Units** | **3974--1** | **3974--1** | **3974--1** | **Mean** | **SD** | **RSD** |
| **1** | **DMP** | **ppb** | **0.13** | **0.11** | **0.13** | **0.12** | **0.01** | **9.4** |
| **2** | **DEP** | **ppb** | **0.29** | **0.27** | **0.25** | **0.27** | **0.02** | **6.5** |
| **3** | **IBP** | **ppb** | **2.61** | **2.53** | **2.43** | **2.52** | **0.09** | **3.4** |
| **4** | **DBP** | **ppb** | **0.48** | **0.47** | **0.46** | **0.47** | **0.01** | **2.2** |
| **5** | **BBP** | **ppb** | **0.10** | **0.09** | **0.09** | **0.11** | **0.01** | **5.2** |
| **6** | **BEHP** | **ppb** | **4.14** | **4.30** | **4.21** | **4.22** | **0.08** | **1.9** |
| **7** | **DOP** | **ppb** | **0.39** | **0.41** | **0.41** | **0.40** | **0.01** | **2.7** |

**Table S3. Identifying, quantifying ions, retention time, and collision energy of 7 phthalates**

| **Compound** | **Abbreviations** | **Retention time (min)** | **Confirmation ions (m/z)** | **Quantification ion (m/z)** |
| --- | --- | --- | --- | --- |
| **Di methyl phthalate** | **DMP** | **13.4** | **77,135, 163, 194** | **163** |
| **Di ethyl phthalate** | **DEP** | **15.8** | **121, 149, 177, 222** | **149** |
| **Di buthylphthalate** | **DBP** | **21.3** | **121, 149, 205, 223** | **149** |
| **bis(2-ethylhexyl) phthalate** | **BEHP** | **26.3** | **45, 72, 121, 149** | **149** |
| **butyl benzyl phthalate** | **BBP** | **28.5** | **91,149, 206, 238** | **149** |
| **di n-octyl-phthalate** | **DNOP** | **30.4** | **149,179,261, 79** | **149** |
