# Supplementary material for: Occurrence, distribution and risk assessment of phthalate esters in dust deposited in the outdoor environment of Yazd industrial park using Monte Carlo simulation
Source: Heliyon. 2024 Sep 8;10(18):e37500. doi: 10.1016/j.heliyon.2024.e37500 (PMC11416271; doi:10.1016/j.heliyon.2024.e37500)
Supplement: Multimedia component 2 [file mmc2.docx]

**
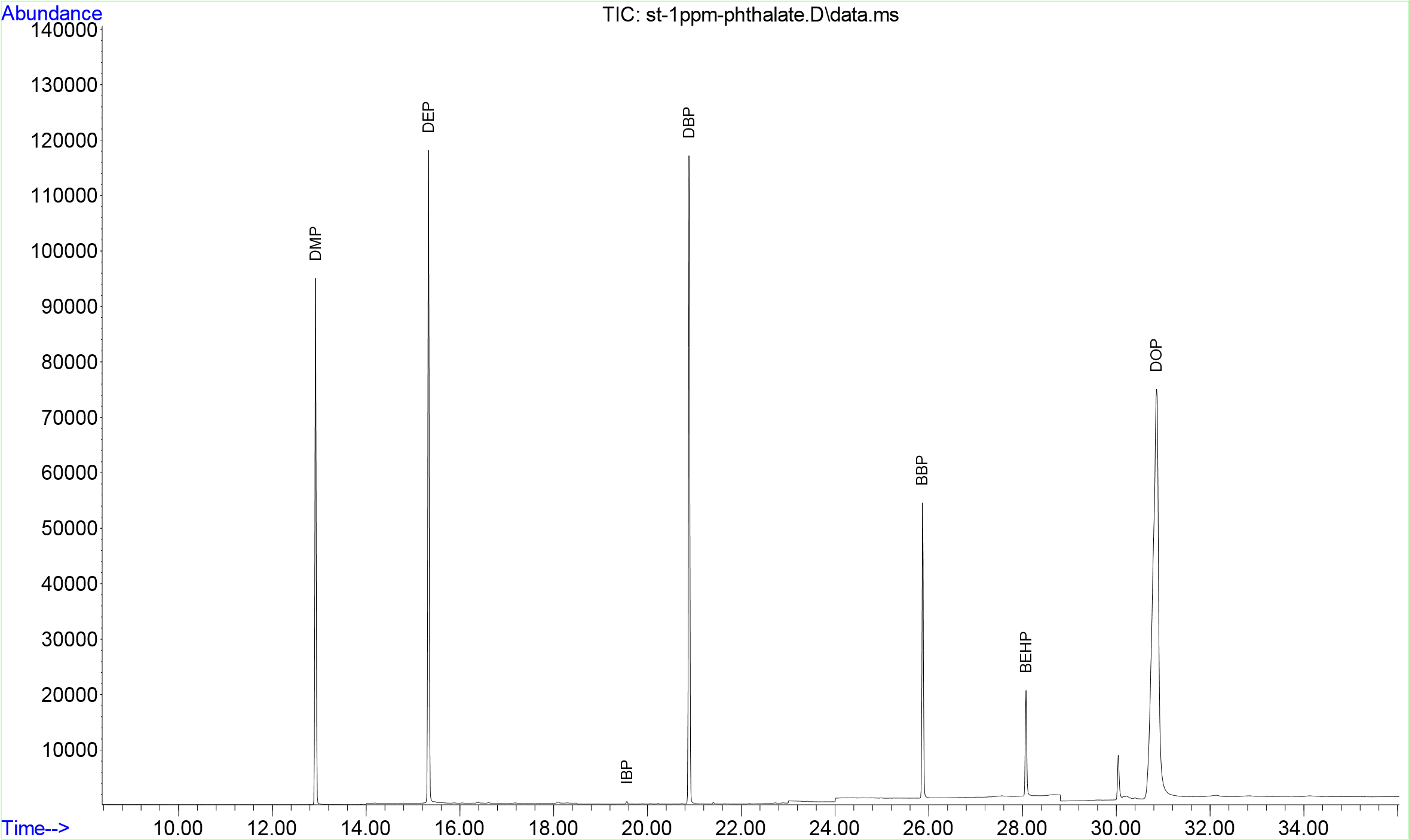
**

Fig. 1S. Chromatogram of different PEs in one of the samples dusts using Acq Method Phthalate-Perfume.


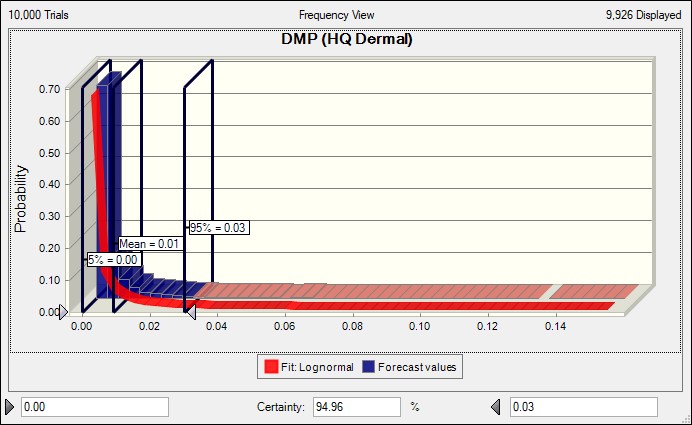

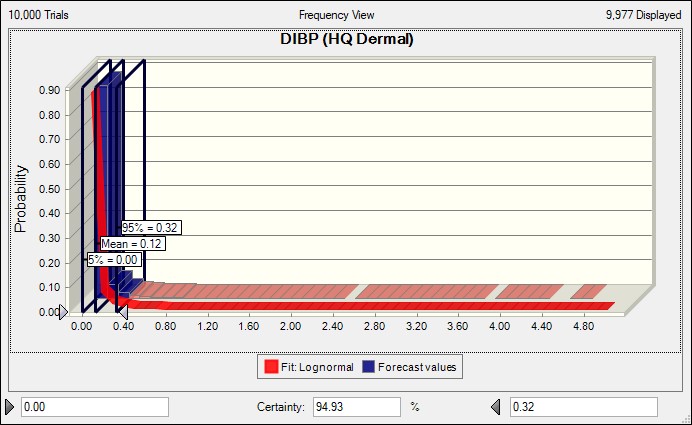


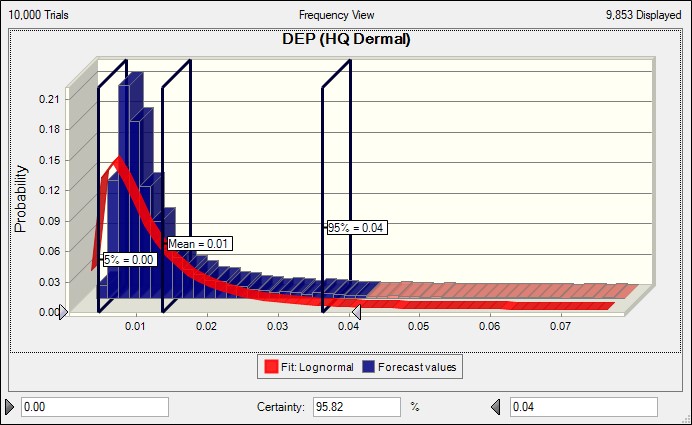

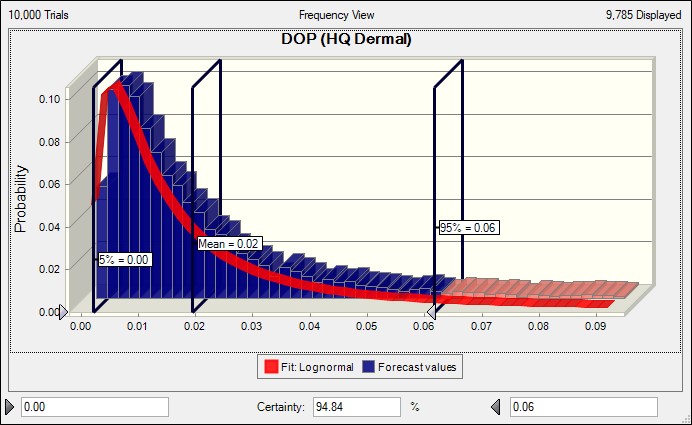


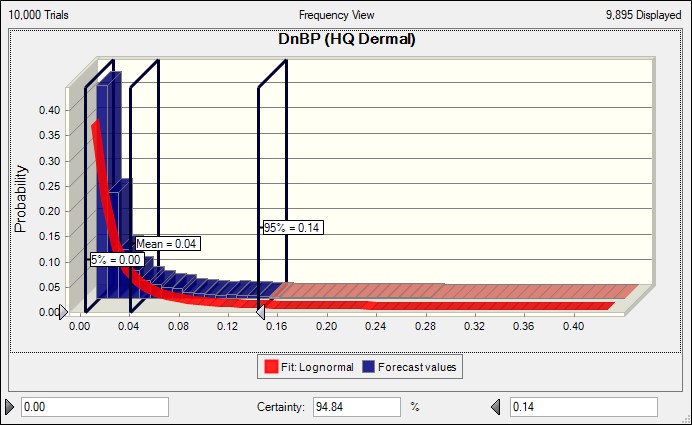


**Figures S2.** Probability Distribution of DMP, DIBP, DEP, DOP and DnBP Inhalation Hazard Quotients Using Monte Carlo Simulation in Crystal Ball


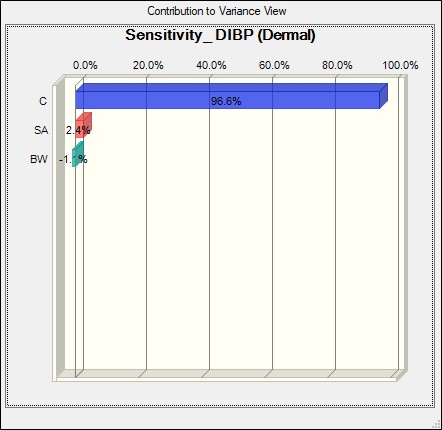

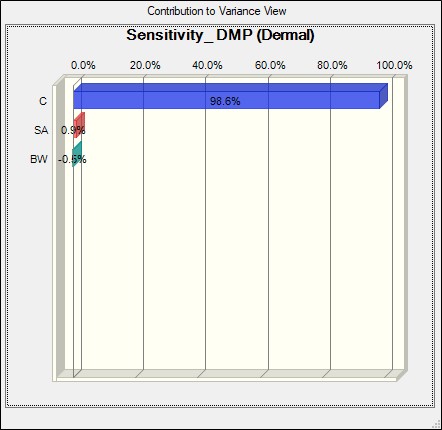


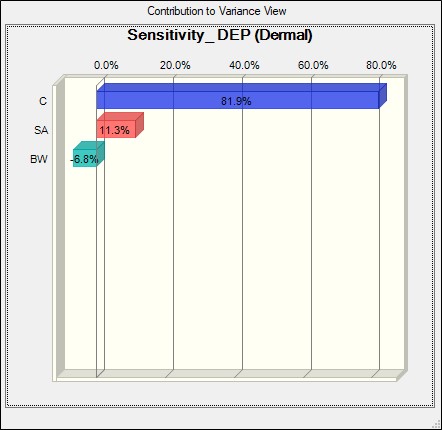

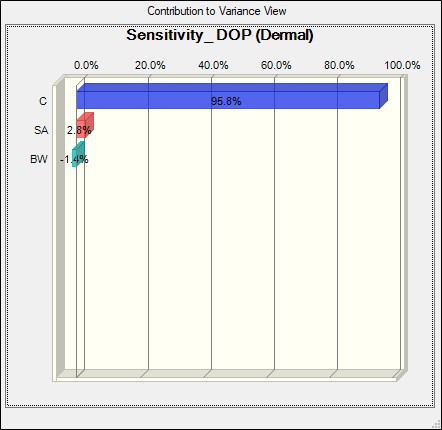


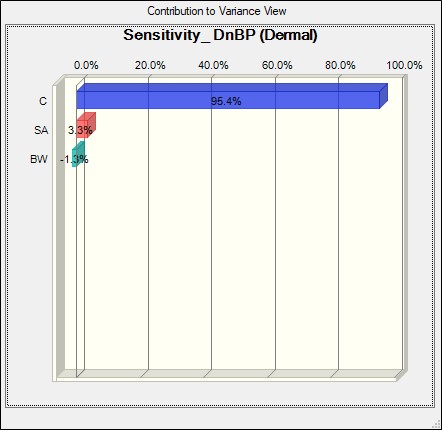


**Figures S3** Sensitivity Analysis of DIBP, DMP, DEP, DOP and DnBP (Inhalation) Using Monte Carlo Simulation in Crystal Ball
